# The complete mitochondrial genome of two different colour *Luciobarbus capito* (Cypriniformes, Cyprinidae)

**DOI:** 10.1080/23802359.2018.1483753

**Published:** 2018-07-27

**Authors:** Shu-Ren Zhu, Yong-An Zhu, Qing-Lei Meng, Li An, Long-Gang Zhang, Zhen-Hai Yu

**Affiliations:** Shandong Provincial Key Laboratory of Freshwater Genetics and Breeding, Shandong Freshwater Fisheries Research Institute, Shandong, China

**Keywords:** *Luciobarbus capito*, different colour, complete mitochondrial genome

## Abstract

We sequenced and characterized the complete mitochondrial genome from normal colour (grey black) and mutant colour (orangey red) of *Luciobarbus capito*. Both mitogenomes contained the typical complement of 13 protein-coding genes, 22 transfer RNAs (tRNAs), two ribosomal RNAs (rRNAs), and a non-coding control region. They share the same gene arrangement pattern that was identical with most vertebrates. The entire mitochondrial DNA molecule of grey black *L. capito* was 16603-bp long, while the complete mtDNA molecule of orangey red *L. capito* was 16607-bp long.

*Luciobarbus capito* belongs to the family Cyprinidae, which originates from Uzbekistan. Because of its fast growth, delicious meat, salt, and alkali resistance, it was introduced to China in 2003. At present, *L. capito* has been successfully promoted in Hebei Province, Jiangsu Province, Shandong Province, and other places, with considerable economic benefits, and has been recognized by the market. In the breeding process, there are normal colour (grey black) and mutant colour (orangey red) of *L. capito*.

In this study, the complete mitochondrial genome of grey black *L. capito* and orangey red *L. capito* were amplified and sequenced. The fish samples were collected from Shandong Freshwater Fisheries Research Institute, China (36°41′10″ latitude, 116°51′3″ longitude) and are deposited in Shandong Provincial Key Laboratory of Freshwater Genetics and Breeding with identifier DLB-O and DLB-B. The complete mitochondrial genome was amplified using 20 primer pairs, which were designed using Primer Premier 5.0 based on *L. capito* (GenBank accession number JX987313). PCR products were sequenced by Map Biotechnology Co., Ltd. The mitogenome of grey black *L. capito* was 16603-bp long, which was slightly shorter than orangey red *L. capito* (16607 bp). All newly determined sequences from the present study were deposited in GenBank database (MH136825 and MH136824).

The structural organization and location of different feature in these mt genomes conformed to the common vertebrate mt genome model and consisted of 13 protein-coding genes, 2 rRNAs, 22 tRNAs, and 1 putative control region (Liu and Cui 2009). Like other vertebrates, most of the genes of *L. capito* were encoded on the H-strand, with only ND6 and eight tRNAs (*Gln*, *Ala*, *Asn*, *Cys*, *Tyr*, *Ser*, *Glu*, and *Pro*) located on the L-strand, and all genes were similar in length to those in other bony fishes (Miya et al. 2003). The gene order was identical to that obtained for other vertebrates. Nucleotide base composition of two complete sequences was the following: 31.25% for A, 27.84% for C, 16.52% for G, 24.39% for T in grey black *L. capito and* orangey red *L. capito.* Except for COX1 with a GTG start codon, the remaining 12 PCGs start with an ATG codon. Different numbers of TA repeats occur in CR for grey black *L. capito and* orangey red *L. capito*, which maybe a potential microsatellite molecular maker.

Phylogenetic analysis was performed by MEGA 6.06 (Tamura et al. 2013) based on the concatenated nucleotide sequences of the 12 protein-coding genes (except ND6) from the mitogenomes of *L. capito* and those of nine closely related species belonging to four genus *Luciobarbus*, *Capoeta*, *Barbus*, and *Spinibarbus*. The maximum-likelihood tree (Figure 1) showed that *L. capito* first clustered together with *L. rifensis*, *L. sclateri*, and formed the genus *Luciobarbus*, and then they constituted a sister-group relationship with other three genuses.

**Figure 1.**
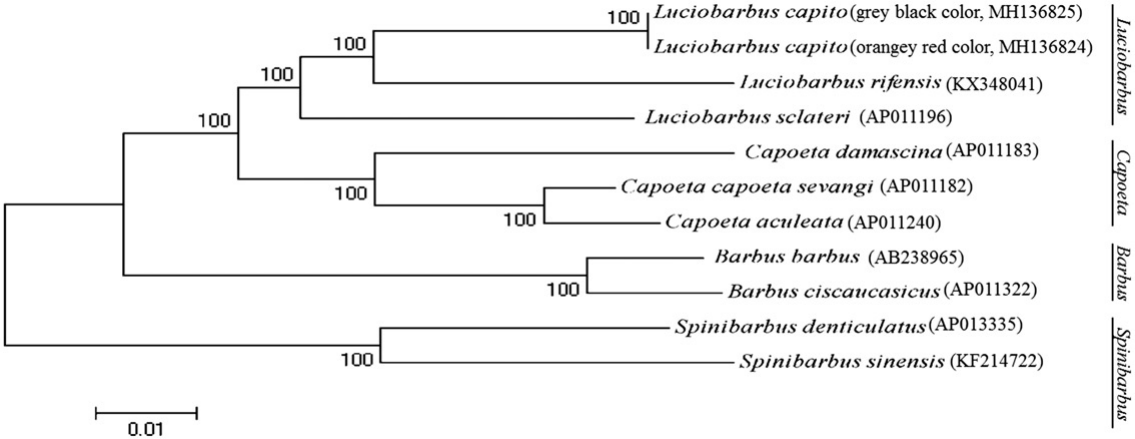
Maximum likelihood (ML) phylogenetic trees inferred from the nucleotide sequence data of mitogenomic 12 protein-coding genes (except ND6).
